# Mapping Community Determinants of Heat Vulnerability

**DOI:** 10.1289/ehp.0900683

**Published:** 2009-06-10

**Authors:** Colleen E. Reid, Marie S. O’Neill, Carina J. Gronlund, Shannon J. Brines, Daniel G. Brown, Ana V. Diez-Roux, Joel Schwartz

**Affiliations:** 1 Environmental Health Sciences Division, School of Public Health, University of California at Berkeley, California, USA; 2 School of Public Health and; 3 School of Natural Resources and the Environment, University of Michigan, Ann Arbor, Michigan, USA; 4 Harvard University School of Public Health, Boston, Massachusetts, USA

**Keywords:** climate, environmental health, geographic information systems, heat, public health, vulnerable populations

## Abstract

**Background:**

The evidence that heat waves can result in both increased deaths and illness is substantial, and concern over this issue is rising because of climate change. Adverse health impacts from heat waves can be avoided, and epidemiologic studies have identified specific population and community characteristics that mark vulnerability to heat waves.

**Objectives:**

We situated vulnerability to heat in geographic space and identified potential areas for intervention and further research.

**Methods:**

We mapped and analyzed 10 vulnerability factors for heat-related morbidity/mortality in the United States: six demographic characteristics and two household air conditioning variables from the U.S. Census Bureau, vegetation cover from satellite images, and diabetes prevalence from a national survey. We performed a factor analysis of these 10 variables and assigned values of increasing vulnerability for the four resulting factors to each of 39,794 census tracts. We added the four factor scores to obtain a cumulative heat vulnerability index value.

**Results:**

Four factors explained > 75% of the total variance in the original 10 vulnerability variables: *a*) social/environmental vulnerability (combined education/poverty/race/green space), *b*) social isolation, *c*) air conditioning prevalence, and *d*) proportion elderly/diabetes. We found substantial spatial variability of heat vulnerability nationally, with generally higher vulnerability in the Northeast and Pacific Coast and the lowest in the Southeast. In urban areas, inner cities showed the highest vulnerability to heat.

**Conclusions:**

These methods provide a template for making local and regional heat vulnerability maps. After validation using health outcome data, interventions can be targeted at the most vulnerable populations.

Exposure to extreme heat can overwhelm a person’s ability to thermoregulate, resulting in physiologic heat stress, which sometimes leads to death (Luber et al. 2006). Studies of heat waves and mortality in the United States demonstrate that days with increased temperature or periods of extended high temperatures have increased heat-related mortality (Chestnut et al. 1998), cardiovascular-cause mortality (Curriero et al. 2002; Medina-Ramon et al. 2006; Semenza et al. 1996), respiratory mortality (Mastrangelo et al. 2007), heart attacks (Braga et al. 2002), and all-cause mortality (Curriero et al. 2002). During heat waves, calls to emergency medical services (Dolney and Sheridan 2006) and hospital admissions (Mastrangelo et al. 2007) also increase.

The Intergovernmental Panel on Climate Change (IPCC) reports that heat waves increased toward the end of the 20th century and are projected to continue to increase in frequency, intensity, and duration worldwide (IPCC 2007), which could result in future increases in heat-related morbidity and mortality. However, heat-related deaths are preventable (Luber et al. 2006). Several cities have implemented heat emergency response plans, and mortality has decreased during subsequent heat waves (Ebi et al. 2004). But many elderly residents in four cities with heat wave warning systems reported that they did not take recommended actions during heat waves (Sheridan 2007), implying that interventions for the most vulnerable populations need improvement. Because not all populations are at equal health risk from heat, knowing where vulnerable populations are located can aid cities in targeting their resources most effectively and, at the state and regional scale, can facilitate coordination of heat emergency plans. A national map of county-level heat vulnerability allows us to situate vulnerability to heat in geographic space and identify areas most in need of intervention.

Although understanding vulnerability to heat at the individual biomedical level is important, understanding also how factors beyond individuals, including “place,” contribute to differing levels of risk may help in finding preventive solutions (Diez Roux 2004; Martinez et al. 1989; Smoyer 1998). Group-level variables (e.g., average income in a census tract) can influence health, independent of the influence of the same variable measured at the individual level (e.g., an individual’s personal or household income) (Diez Roux 2004; Smoyer 1998). Our vulnerability maps include data on both community properties (e.g., low green space) and population composition (e.g., high numbers of elderly residents) that may lead to vulnerability to heat.

The published literature on mapping heat vulnerability is scant. Vescovi et al. (2005) geographically overlaid climate variables with socioeconomic variables in southern Quebec to estimate current vulnerable populations and then estimated future population vulnerability using climate and population projections. Overall, that study projected that the population at risk will increase. Harlan et al. (2006) investigated physical attributes of the environment, socioeconomic characteristics, and an outdoor human thermal comfort index in Phoenix and found that neighborhoods with the highest temperatures and the least amount of open space and vegetation were also the most socioeconomically disadvantaged. A recent publication mapped many heat vulnerability variables by county for the state of California (Climate Change Public Health Impacts Assessment and Response Collaborative 2007). However, they did not make an index or analyze the collocations of their vulnerability variables. All three studies attempted to situate vulnerability in space, but at different spatial scales and with different variables.

Our study expands heat vulnerability mapping to a national scope, using variables shown in the epidemiologic literature to increase vulnerability to heat-related health effects in urban areas. Our goal is to create a cumulative heat vulnerability index for nationwide comparison.

## Methods

### Vulnerability data sources

Table 1 lists the data sources, vulnerability variables, and level of aggregation of the data sets that were used in our analysis. We chose 10 variables that have been demonstrated to modify the relationship between heat and health outcomes in the literature and for which national data sets were available as detailed below. The true nature of some of the associations among these variables and whether effect modification is consistently present are still open to question because not all previous studies investigated the possibility of both confounding and effect modification by various vulnerability variables. However, the weight of available evidence and general plausibility pointed toward these 10 variables as relevant to heat vulnerability. We used a factor analysis to deal with potential multicollinearities. All 10 variables were calculated so that an increase in value denotes an increase in vulnerability.

#### Demographic and socioeconomic variables

The demographic and socioeconomic variables investigated included age, poverty, education, living alone, and race/ethnicity. Age is a vulnerability factor for heat waves because the very old have shown higher mortality during heat waves (Conti et al. 2005; Fouillet et al. 2006; Hutter et al. 2007; Naughton et al. 2002; Stafoggia et al. 2008; Whitman et al. 1997), higher rates of temperature-related deaths in temperature variability studies [Centers for Disease Control and Prevention (CDC) 2001; Kim and Joh 2006; Medina-Ramon et al. 2006], and higher hospital admission rates during heat waves (Knowlton et al. 2009; Semenza et al. 1999). However, not all studies found an increased risk for elderly U.S. residents (O’Neill et al. 2003).

Poverty- and income-related variables also modify the effects of heat in some studies. Community levels of poverty modified associations between heat and mortality for 11 eastern U.S. cities (Curriero et al. 2002). A modest increase in risk of heat-related death was observed for those making less than versus more than $10,000 during the 1999 Chicago heat wave (Naughton et al. 2002). In Seoul, Korea, people of low income had higher mortality rates during hot weather (Kim and Joh 2006).

Individuals with at most a high school education had higher heat-related death rates than did those with more years of education in studies of seven U.S. cities (O’Neill et al. 2003) and fifty U.S. cities (Medina-Ramon et al. 2006). In studies of area-level indicators of educational level, no significant effect modification was found for attained high school education in nine California counties (Basu and Ostro 2008), or for the percentage of residents of each city with a college education in 12 U.S. cities (Braga et al. 2002). The percentage of residents in each city with a high school education, however, did modify the heat-mortality relationship for 11 eastern U.S. cities (Curriero et al. 2002).

A sociologic analysis of the 1995 Chicago heat wave found that large numbers of the victims of the heat wave died alone (Klinenberg 2003), and an epidemiologic study of the same heat wave found that people who did not leave home each day or who lived alone had a higher risk of death compared with people with social contacts and access to transportation (Semenza et al. 1996). Similar results were found for the 1999 Chicago heat wave (Naughton et al. 2002). However, living alone did not modify the risk of heat-related death in Modena, Italy (Foroni et al. 2007), or in England and Wales (Hajat et al. 2007). Married people were less likely to die from heat compared with those who were widowed, divorced, or never married in both Italy (Stafoggia et al. 2008) and France (Fouillet et al. 2006). Although not all people who are single, widowed, or divorced live alone, this may be a proxy for either living alone or not being checked on regularly during a heat emergency.

Comparisons of heat-related deaths by racial or ethnic groups show mixed results. The CDC found that blacks had a higher age-adjusted heat-related death rate than did whites throughout the United States from 1979 through 1998 (CDC 2001). Kalkstein and Davis (1989) found strong correlations between heat mortality and percent non-white only in southern U.S. cities (Kalkstein and Davis 1989). In Detroit, nonwhites had a higher risk of death on hot days (O’Neill et al. 2003; Schwartz 2005), and during the 1995 Chicago heat wave, non-Hispanic blacks had higher death rates than did non-Hispanic whites (Whitman et al. 1997). In a case– control study, blacks had a higher death rate than did whites; however, both had higher rates than did Hispanics (Basu and Ostro 2008). Modification of the relationship between heat and mortality by race, however, has not been found in all studies (Braga et al. 2002). Differential mortality rates may be partially explained by differences in air conditioning (AC) prevalence in homes by race. Sixty-four percent of the disparity in heat-related mortality between blacks and whites from four U.S. cities may be explained by the prevalence of central AC in homes (O’Neill et al. 2005).

The 2000 U.S. Census demographic variables age, poverty, education, living alone, and race/ethnicity were extracted from the Planner’s Package Plus data product from Geolytics, Inc. (East Brunswick, NJ) and aggregated at the census tract level for all tracts within the coterminous United States.

#### Land cover

The existence of green space in a community has been associated with a decreased risk of heat-related illness and death. A case–control study of the 1980 heat wave in St. Louis and Kansas City, Missouri, found a significant decrease in risk of nonfatal heat-stroke associated with an incremental increase in greenery surrounding residences (Kilbourne et al. 1982). In Shanghai, a decrease in deaths in the 2003 heat wave compared with the 1998 heat wave was partially attributed to an increase in urban green area (Tan et al. 2007). Urban areas tend to have less green space and more impervious cover, which contribute to the urban heat island effect. This can further exacerbate a heat wave, and the higher city death rates during the 1966 St. Louis heat wave were hypothesized to be due to the hotter temperatures in the city (Clarke 1972). A national map of heat-related deaths in the elderly from 1979 through 1985 found that most of the high-incidence areas were urban counties (Martinez et al. 1989), and in England and Wales, the relative risk of death was higher in urban than in rural areas (Hajat et al. 2007).

We downloaded the 2001 National Land Cover Database (NLCD) for the coterminous United States (U.S. Department of the Interior 2008) and aggregated data at the census tract level by overlaying census tract polygons on the classified imagery. Each 30-m pixel from the NLCD was assigned to the census tract polygon in which its center was located. Percent green space for each census tract was calculated as the sum of land area classified as deciduous forest, evergreen forest, mixed forest, dwarf scrub, orchards/vineyards/other, pasture/hay, small grains, fallow, row crops, urban/recreational grasses, palustrine forested wetlands, and palustrine scrub/shrub wetlands divided by the total area for that census tract. Percent “not green space” was calculated as 100 minus the percent green space.

#### Diabetes prevalence

Preexisting health conditions may lead to susceptibility to heat-related illnesses and death. These conditions include cardiovascular disease (Naughton et al. 2002; Semenza et al. 1996, 1999; Stafoggia et al. 2006); diabetes (Schwartz 2005; Semenza et al. 1999); renal disease, nervous disorders, emphysema, and epilepsy (Semenza et al. 1999); cerebrovascular disease (Stafoggia et al. 2006, 2008); pulmonary conditions (Semenza et al. 1996); and mental health conditions (Foroni et al. 2007; Semenza et al. 1996; Stafoggia et al. 2006, 2008). However, for most of these variables, a consistent national data set of prevalence does not currently exist, so we were only able to map diabetes prevalence.

We calculated diabetes prevalence from the 2002 Behavioral Risk Factor Surveillance System (BRFSS; U.S. Department of Health and Human Services 2008) state prevalence rates, which are reported by age, race, and gender groups. From the 2000 U.S. Census data from the Planners’ Package Plus, we obtained population estimates for each age and race and multiplied these by the BRFSS state diabetes rate for that age and racial group, obtaining an estimate of diabetes cases for that group in that county. We then summed these values for all age and race groups in the county and divided by the county population to achieve an estimate of diabetes prevalence for each county in each state. Correlation between our county-level estimates and the few metropolitan statistical area (MSA) estimates published by BRFSS was good (*R*^2^ = 0.72).

#### Air conditioning

Home AC prevalence can be a strong protective factor against heat-related deaths (Braga et al. 2001; Curriero et al. 2002; Kaiser et al. 2001; Naughton et al. 2002; Semenza et al. 1996). Although both room and central AC had negative correlations with heat-related mortality (Chestnut et al. 1998), central AC may have a stronger protective effect than room AC (Chestnut et al. 1998; O’Neill et al. 2003).

AC prevalence data were collected from both the metropolitan area and national surveys of the U.S. Census Bureau’s American Housing Survey (AHS; U.S. Census Bureau 2008) for all counties (*n* = 464) for which the MSA is indicated in the source data. We calculated the percentage of households with central AC and with any AC by county, either as a direct estimate for the year 2002 or as an interpolation from values for neighboring years, because the AHS survey is administered in different MSAs in different years.

### Analysis

We obtained census-tract–level data for all variables except diabetes and AC prevalence, which we assigned to census tracts from county data because data for smaller areas were not available. We selected only the census tracts for which we had data for all variables, limiting us to counties with data from the AHS (i.e., urban areas; *n* = 41,043). We then restricted our analysis to census tracts with populations of at least 1,000 people (*n* = 39,794).

Spearman’s correlation coefficients were calculated between the 10 vulnerability variables. We then used principal components analysis to limit the number of variables and create independent factors for inclusion in a vulnerability index. A varimax rotation was used to minimize the number of the original variables that load highly on any one factor and increase the variation among factors, thus making these new factors more statistically independent than the original variables. We retained four factors based on a combination of standard criteria: eigenvalues > 1, a clear break in values in the scree test, and the percentage of variance explained by the factors. Factor scores were calculated for each of the four factors for each census tract using estimated scoring coefficients based on the factor analysis in SAS (version 9.1; SAS Institute Inc., Cary, NC).

The calculated factor scores were normalized to have a mean of 0 and a standard deviation of 1. For ease of interpretation and to minimize the impact of outliers, we divided each factor into six categories based on standard deviations, as shown in Table 2. We assigned scores to each category, with 1 corresponding to the lowest vulnerability and 6 to the highest. Because we have no knowledge of nonlinearities in these relationships, we assumed linear relationships between each variable and vulnerability. In the absence of detailed understanding of the impacts of each factor on vulnerability, we assumed they each had equal impact and summed the assigned factor values for the four factors, creating a cumulative heat vulnerability index value for each census tract.

Because heat may influence health differently depending on prevailing climate conditions, because of physiologic and structural adaptations, we calculated the mean apparent temperature for MSAs from 1985 to 2003 and assessed whether there was a significant relationship between this value and the cumulative heat vulnerability index.

## Results

Many of the 10 vulnerability variables were highly correlated, as shown in Table 3. Factor analysis yielded four factors with primary loadings: *a*) social/environmental vulnerability (combined education/poverty/proportion people of color/green space), *b*) social isolation, *c*) prevalence of no AC, and *d*) proportion elderly/with diabetes. These four factors explained 75.7% of the variability in the original 10 vulnerability variables, as shown in Table 4.

**Figure 1:**
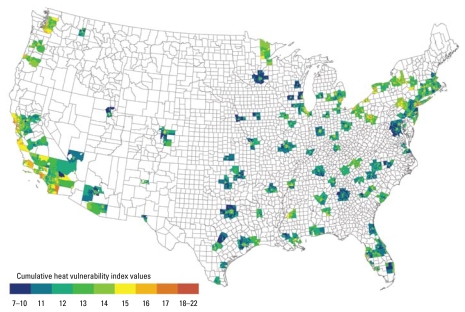
National map of cumulative heat vulnerability index by census tract (*n* = 39,794).

The cumulative heat vulnerability index values, summed from the four factors for each census tract, ranged from 7 to 22, with a mean of 13.94, a median of 14, and an SD of 2.02. The 39,794 census-tract–level cumulative vulnerability index values were fairly normally distributed. Figure 1 shows the national geographic distribution of the cumulative vulnerability index, with evidence of spatial clustering. Overall, higher vulnerability was seen in the Northeast and along the Pacific Coast, with some pockets of higher vulnerability in the Southeast and along the U.S.–Mexico border. Thirteen census tracts had the highest cumulative heat vulnerability index values (21 or 22). Eight of these are in the San Francisco Bay Area (San Francisco County and Alameda County); two are in Cuyahoga County, Ohio; one is in Pierce County, Washington; and one is in Los Angeles County, California. All of these census tracts are above the mean for all four factors. No census tract reached the highest vulnerability category for all four factors.

We then calculated each MSA’s mean cumulative heat vulnerability index value and ordered them from lowest to highest, looking at the contributions of each factor to the overall vulnerability. Only factor 3, prevalence of no AC, appeared to increase as the cumulative index increased (data not shown). To check whether AC was driving our vulnerability index, we did a factor analysis of vulnerability variables without AC and found that this yielded only three retained vulnerability factors, without the factor for AC as expected. Further analysis showed that although this did decrease vulnerability for the Pacific Coast and the Northeast, the changes were minimal. Also, almost equal numbers of tracts showed increases as showed decreases. Although this may imply that lack of AC is driving our vulnerability index, not all areas with low AC prevalence had high cumulative heat vulnerability values. Additionally, the importance of AC use in protecting against heat-related health outcomes is clear. Therefore, removing it from our index would not improve our estimates of vulnerability.

Mean MSA values also highlight the regional variation in heat vulnerability, with the 20 most vulnerable cities located on the Pacific Coast or Northeast, topped by San Francisco, New York, New York, and Los Angeles. However, the 20 least vulnerable cities, although mostly in the Southeast (e.g., Austin, TX; Atlanta, GA; and Raleigh-Durham, NC), do include some cities from the Northeast and Midwest. For example, Minneapolis, Minnesota, was the third least vulnerable MSA, and smaller MSAs in Massachusetts and New Jersey also fell in the 10 least vulnerable cities.

Analysis by climatic region did not provide evidence for a trend in cumulative heat vulnerability by the mean apparent temperature from 1983 through 2003 by MSA (data not shown). The only individual factor that showed a trend with apparent temperature was factor 3, prevalence of no AC, which decreased with increasing apparent temperature, as expected. However, the relationship was not very strong.

**Figure 2:**
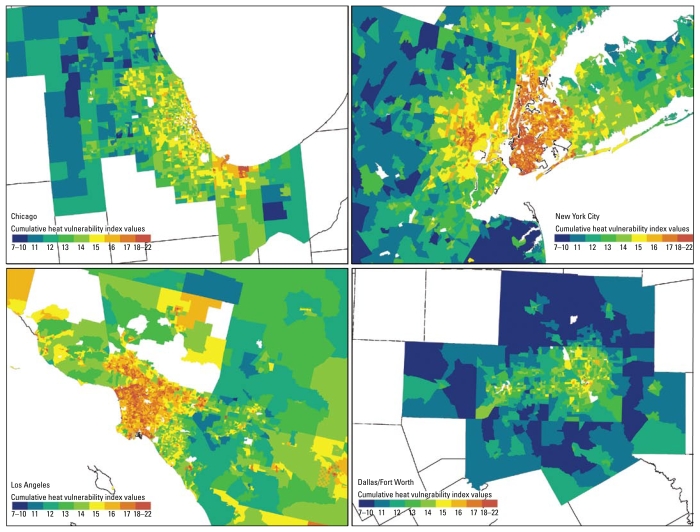
Mean cumulative heat vulnerability maps by census tract for four selected cities.

Figure 1 illustrates the national variability in heat vulnerability and variation within cities. In most cities, including those where most areas have low heat vulnerability, the downtown areas show the most vulnerability (Figure 2). For example, although Dallas shows less overall heat vulnerability than do the other cities in Figure 2, it contains areas of higher vulnerability in its central area. This pattern was found in many other low-vulnerability Southeast and Midwest cities. Local spatial autocorrelation analysis for individual MSAs showed significant clustering of high vulnerability in downtown areas and clustering of low vulnerability in outlying areas.

## Discussion

In our analysis of urban areas in the United States, heat vulnerability varies nationally and is concentrated in central city areas. Epidemiologic studies are increasingly assessing vulnerability of specific populations and geographic areas to heat waves. We used knowledge from previous epidemiologic research to develop a map that can be used to focus interventions to prevent heat-related morbidity and mortality and to suggest directions for future research. Our analysis is an approach similar to methodologies used to map social vulnerability to environmental hazards (Cutter et al. 2003).

Epidemiologic studies investigating different geographic regions in the same study have also found regional differences in response to heat (Basu et al. 2008; Conti et al. 2007; Curriero et al. 2002; Hajat et al. 2007; O’Neill et al. 2003), possibly due to ways in which the populations of those cities have adapted physiologically, socially, and/or technologically to heat. However, most of these studies, when assessing modification of the heat–health relationship by vulnerability variables such as those used in this analysis (e.g., race, educational attainment), pool nationwide data rather than comparing vulnerabilities among regions, O’Neill et al. (2003) being one exception. Increased understanding of differential effect modification by geographic region could be used to further refine our heat vulnerability map.

Of the vulnerability factors created in this analysis, factor 3, prevalence of no AC, showed the most national spatial variability, and regions with the highest AC prevalence had some of the lowest cumulative heat vulnerability values. For example, areas along the West Coast showed very high vulnerability even though their current climates are temperate. In the event of a heat wave, they will likely have significant vulnerability to heat. Efforts should be made to create incentives for people to use their AC during heat waves, because the economic costs of AC use deter people who have AC in their homes from turning it on during a heat wave (Sheridan 2007). Although AC can protect against heat, caution should be applied in promoting AC as the sole heat wave adaptation strategy. AC uses electricity, most of which comes from fossil fuel energy sources, and additionally exhausts waste heat to the local environment, thus increasing the urban heat island effect. Other modifications to the built environment such as tree and shrub planting, reflective paving surfaces, and natural ventilation can reduce heat exposure in a more sustainable manner.

Although our map shows differences in heat vulnerability between regions of the country, it also highlights the higher vulnerability within the downtowns of metropolitan areas. Because heat warning systems and interventions are often implemented at the municipal or local level, identifying these regions within cities is essential. Heat waves can occur in any community, and with climate change, heat waves are projected to increase in frequency, duration, and intensity in the United States (Meehl and Tebaldi 2004). Therefore, municipalities should incorporate heat wave warning systems and interventions into their emergency planning procedures, focusing on ways to improve the compliance in the response, particularly of elderly adults, to such warnings (see Sheridan 2007).

Within-city analyses of heat vulnerability may give more information about local vulnerability than a national map. Also, relationships between variables may be different at smaller spatial scales, resulting in different vulnerability factors and thus different geographic variability. The methodology presented in this article can be used for these local vulnerability maps. Identifying not only the most vulnerable populations in the community but also whether those areas already experience the hottest temperatures, as in Phoenix (Harlan et al. 2006), and ameliorating these local heat hotspots within the urban heat island with cool roofs and urban trees could go a long way toward mitigating heat. All metropolitan areas in this analysis, regardless of AC prevalence, had higher social vulnerabilities in their downtown core that make those areas more vulnerable to many exposures, heat being just one of them. Targeting these inequalities could lead to reductions in many health outcomes.

Our analysis introduces a methodology of vulnerability mapping for heat-related health outcomes that can serve as a template for future heat vulnerability maps at local and regional levels. The use of health data to validate our measures of heat-related vulnerability is an important next step. This could further highlight local or regional differences about which factors contribute most to vulnerability and therefore are important intervention targets. For example, the downtown area of Oakland, with little green space and a high proportion of residents of color and people living in poverty, has not recently been the location of most of the heat-related health effects, possibly because these neighborhoods are located closer to the cooling breezes of San Francisco Bay (English P, personal communication, 2008). Thus, local information is essential for ensuring the validity of this map at local scales. However, at regional and national scales, our map can provide guidance on locations for further analysis and intervention. At a national level, a method for weighting cities according to the probability of a heat wave can help determine which cities are most in need of heat wave intervention programs.

Our analysis was limited by the data available at the national level. Variables of preexisting health concerns that denote vulnerability to heat, such as cardiovascular disease or psychiatric disorders, are not currently available nationally, but may be in the future. Other vulnerability variables are likely to be available only through local surveys, such as degree of social connections among individuals within a community, or materials used in housing. Additionally, some variables such as crime rates merit further investigation as modifiers of heat and health associations in epidemiologic analyses.

We limited our analysis to urban areas, in which most heat wave health effects have occurred and for which we understand more about which conditions make individuals and communities more vulnerable. Sheridan and Dolney (2003), however, found that although higher absolute numbers of heat-related deaths occurred in urban counties in Ohio, the percentage increase in mortality during heat waves was greater in suburban and rural counties, thus highlighting an important area for future research. Rural populations may exhibit patterns of vulnerability different from those of urban populations.

With further information on the degree to which a given vulnerability variable modifies the heat–health relationship, more complex algorithms can be applied to more accurately value heat vulnerability, including differential weighting of the variables we examined or the inclusion of different or additional variables. We assessed whether summing the factor scores without rescaling and weighting them would create a different heat vulnerability map. This allowed for more gradation in vulnerability and finer stratification of regions, but AC prevalence still played a large role in cumulative heat vulnerability, and the same regions of the country and regions of cities showed comparatively higher and lower vulnerability.

## Conclusions

Heat vulnerability varies spatially, on local, regional, national, and international scales. With further validation at the local scale and evaluation with health outcome data, our methodology and results can help target resources for intervention. In our analysis, in addition to regional difference in heat vulnerability, higher vulnerability was seen within the downtown areas of all cities compared with suburban areas regardless of the city’s overall vulnerability.

This study is a novel approach to map vulnerability to a health outcome related to climate change nationally and can be considered a first step toward tools that can help public health professionals prepare climate change adaptation plans for their communities. In addition to refinement of this method for heat vulnerability, further studies mapping vulnerability to other projected health impacts of climate change are needed.

## Figures and Tables

**Table 1:** Heat-health vulnerability data, 39,794 U.S. census tracts.

Category	Data source (year)	Variable definition	Percent mean (range)
Demographic variables	U.S. Census (2000)	Percent population below the poverty line	12.57 (0.00–100.00)
		Percent population with less than a high school diploma	19.97 (0.00–85.88)
		Percent population of a race other than white	30.20 (0.00–100.00)
		Percent population living alone	10.28 (0.00–68.86)
		Percent population ≥ 65 years of age	12.21 (0.00–94.28)
		Percent population ≥ 65 of age living alone	27.38 (0.00–100.00)
Land cover	National Land Cover Database (2001)	Percent census tract area not covered in vegetation	61.15 (0.03–100.00)
Diabetes prevalence	Behavioral Risk Factor Surveillance System (2002)	Percent population ever diagnosed with diabetes	6.95 (2.38–11.10)
Air conditioning	American Housing Survey (2002)^a^	Percent households without central AC	44.43 (2.10–95.13)
		Percent households without any AC	18.47 (0.00–95.13)

a Data were interpolated for 2002 for counties that were surveyed in years before and after 2002 to get a larger sample of air conditioning estimates for 1 year.

**Table 2:** Counts of census tracts for each heat vulnerability factor by categories created by observed distributions.

		No. of census tracts (%)			
Category	Assigned value	Factor 1: social/environmental vulnerability	Factor 2: social isolation	Factor 3:prevalence of no AC	Factor 4: proportion elderly/diabetes
≥ 2 SD below mean	1	64 (0.16)	141 (0.35)	0 (0.00)	670 (1.68)
1–2 SD below mean	2	4,163 (10.46)	4,941 (12.42)	7,567 (19.02)	5,276 (13.26)
< 1 SD below mean	3	20,186 (50.73)	17,296 (43.46)	14,658 (36.83)	14,633 (36.77)
< 1 SD above mean	4	8,117 (20.40)	12,107 (30.42)	10,239 (25.73)	13,617 (34.22)
1–2 SD above mean	5	5,208 (13.09)	3,687 (9.27)	6,136 (15.42)	4,583 (11.52)
> 2 SD above mean	6	2,056 (5.17)	1,622 (4.08)	1,194 (3.00)	1,015 (2.55)

**Table 3:** Spearman’s correlation values for vulnerability variables for census tracts nationwide (*n* = 39,794).

	Diabetes	Race other than white	Age > 65 years	Live alone	Age > 65 living alone	Below poverty line	Less than high school diploma	Not green space	No central AC	No AC of any kind
Diabetes	1.00									
Race other than white	0.25	1.00								
Age ≥ 65 years	0.13	−0.31	1.00							
Live alone	0.07	−0.03	0.47	1.00						
Age ≥ 65 living alone	0.06	0.06	0.22	0.69	1.00					
Below poverty line	0.27	0.64	−0.11	0.22	0.33	1.00				
Less than high school diploma	0.28	0.56	−0.05	−0.02	0.17	0.77	1.00			
Not green space	0.27	0.50	−0.02	0.23	0.24	0.43	0.35	1.00		
No central AC	0.11	−*0.00*	0.09	*0.01*	0.05	−*0.01*	−*0.01*	0.25	1.00	
No AC of any kind	0.11	0.02	−0.03	−0.03	*0.01*	−*0.01*	−0.03	0.25	0.85	1.00

All values are statistically significant at *p* < 0.001 except for those in italics.

**Table 4:** Factor loadings for heat vulnerability variables for the four retained varimax-rotated factors based on data from 39,794 census tracts.

	Factor 1: social/environmental vulnerability	Factor 2: social isolation	Factor 3: prevalence of no AC	Factor 4: proportion of elderly/diabetes
Diabetes	0.37	−0.10	0.07	0.78
Below poverty line	0.87	0.18	−0.05	−0.03
Race other than white	0.85	−0.05	0.03	0.02
Live alone	−0.06	0.91	−0.002	0.16
Age ≥ 65 living alone	0.19	0.87	0.001	−0.06
Age ≥ 65 years	−0.32	0.38	−0.04	0.67
Less than high school diploma	0.85	−0.06	−0.05	0.07
Not green space	0.54	0.33	0.31	0.13
No central AC	0.02	0.02	0.92	0.06
No AC of any kind	−0.01	−0.03	0.92	−0.03

Absolute values > 0.4 are the most significant loadings on that factor.

## References

[b1-ehp-117-1730] Basu R, Feng WY, Ostro BD (2008). Characterizing temperature and mortality in nine California counties. Epidemiology.

[b2-ehp-117-1730] Basu R, Ostro BD (2008). A multicounty analysis identifying the populations vulnerable to mortality associated with high ambient temperature in California. Am J Epidemiol.

[b3-ehp-117-1730] Braga AL, Zanobetti A, Schwartz J (2001). The time course of weather-related deaths. Epidemiology.

[b4-ehp-117-1730] Braga AL, Zanobetti A, Schwartz J (2002). The effect of weather on respiratory and cardiovascular deaths in 12 U.S. cities. Environ Health Perspect.

[b5-ehp-117-1730] CDC (Centers for Disease Control and Prevention) (2001). Heat-related deaths—Los Angeles County, California, 1999–2000, and United States, 1979–1998. JAMA.

[b6-ehp-117-1730] Chestnut LG, Breffle WS, Smith JB, Kalkstein LS (1998). Analysis of differences in hot-weather-related mortality across 44 US metropolitan areas. Environ Sci Policy.

[b7-ehp-117-1730] Clarke JF (1972). Some effects of the urban structure on heat mortality. Environ Res.

[b8-ehp-117-1730] Climate Change Public Health Impacts Assessment and Response Collaborative (2007). Heat-Related Illness and Mortality, Information for the Public Health Network in California.

[b9-ehp-117-1730] Conti S, Masocco M, Meli P, Minelli G, Palummeri E, Solimini R (2007). General and specific mortality among the elderly during the 2003 heat wave in Genoa (Italy). Environ Res.

[b10-ehp-117-1730] Conti S, Meli P, Minelli G, Solimini R, Toccaceli V, Vichi M (2005). Epidemiologic study of mortality during the summer 2003 heat wave in Italy. Environ Res.

[b11-ehp-117-1730] Curriero FC, Heiner KS, Samet JM, Zeger SL, Strug L, Patz JA (2002). Temperature and mortality in 11 cities of the eastern United States. Am J Epidemiol.

[b12-ehp-117-1730] Cutter SL, Boruff BJ, Shirley WL (2003). Social vulnerability to environmental hazards. Soc Sci Q.

[b13-ehp-117-1730] Diez Roux AV (2004). The study of group-level factors in epidemiology: rethinking variables, study designs, and analytical approaches. Epidemiol Rev.

[b14-ehp-117-1730] Dolney TJ, Sheridan SC (2006). The relationship between extreme heat and ambulance response calls for the city of Toronto, Ontario, Canada. Environ Res.

[b15-ehp-117-1730] Ebi KL, Teisberg TJ, Kalkstein LS, Robinson L, Weiher RF (2004). Heat watch/warning systems save lives. B Am Meteorol Soc.

[b16-ehp-117-1730] Foroni M, Salvioli G, Rielli R, Goldoni CA, Orlandi G, Sajani SZ (2007). A retrospective study on heat-related mortality in an elderly population during the 2003 heat wave in Modena, Italy: the Argento Project. J Gerontol A Biol Sci Med Sci.

[b17-ehp-117-1730] Fouillet A, Rey G, Laurent F, Pavillon G, Bellec S, Ghihenneuc-Jouyaux C (2006). Excess mortality related to the August 2003 heat wave in France. Int Arch Occup Environ Health.

[b18-ehp-117-1730] Hajat S, Kovats RS, Lachowycz K (2007). Heat-related and cold-related deaths in England and Wales: who is at risk?. Occup Environ Med.

[b19-ehp-117-1730] Harlan SL, Brazel AJ, Prashad L, Stefanov WL, Larsen L (2006). Neighborhood microclimates and vulnerability to heat stress. Soc Sci Med.

[b20-ehp-117-1730] Hutter HP, Moshammer H, Wallner P, Leitner B, Kundi M (2007). Heatwaves in Vienna: effects on mortality. Wien Klin Wochenschr.

[b21-ehp-117-1730] Solomon S, Qin D, Manning M, Chen Z, Marquis M, Averyt KB, Tignor M, Miller HL, IPCC (2007). Summary for policymakers. Climate Change 2007: The Physical Science Basis Contribution of Working Group I to the Fourth Assessment Report of the Intergovernmental Panel on Climate Change.

[b22-ehp-117-1730] Kaiser R, Rubin CH, Henderson AK, Wolfe MI, Kieszak S, Parrott CL (2001). Heat-related death and mental illness during the 1999 Cincinnati heat wave. Am J Forensic Med Pathol.

[b23-ehp-117-1730] Kalkstein LS, Davis RE (1989). Weather and human mortality: an evaluation of demographic and interregional responses in the United States. Ann Assoc Am Geogr.

[b24-ehp-117-1730] Kilbourne EM, Choi K, Jones S, Thacker SB (1982). Risk factors for heatstroke. A case-control study. JAMA.

[b25-ehp-117-1730] Kim Y, Joh S (2006). A vulnerability study of the low-income elderly in the context of high temperature and mortality in Seoul, Korea. Sci Total Environ.

[b26-ehp-117-1730] Klinenberg E (2003). Review of heat wave: a social autopsy of disaster in Chicago. N Engl J Med.

[b27-ehp-117-1730] Knowlton K, Rotkin-Ellman M, King G, Margolis HG, Smith D, Solomon G (2009). The 2006 California heat wave: impacts on hospitalizations and emergency department visits. Environ Health Perspect.

[b28-ehp-117-1730] Luber G, Sanchez C, Conklin L (2006). Heat-related deaths—United States, 1999–2003. MMWR Morb Mortal Wkly Rep.

[b29-ehp-117-1730] Martinez BF, Annest JL, Kilbourne EM, Kirk ML, Lui K-J, Smith ZM (1989). Geographic distribution of heat-related deaths among elderly persons. Use of county-level dot maps for injury surveillance and epidemiologic research. JAMA.

[b30-ehp-117-1730] Mastrangelo G, Fedeli U, Visentin C, Milan G, Fadda E, Spolaore P (2007). Pattern and determinants of hospitalization during heat waves: an ecologic study. BMC Public Health.

[b31-ehp-117-1730] Medina-Ramon M, Zanobetti A, Cavanagh DP, Schwartz J (2006). Extreme temperatures and mortality: assessing effect modification by personal characteristics and specific cause of death in a multi-city case-only analysis. Environ Health Perspect.

[b32-ehp-117-1730] Meehl GA, Tebaldi C (2004). More intense, more frequent, and longer lasting heat waves in the 21st Century. Science.

[b33-ehp-117-1730] Naughton MP, Henderson A, Mirabelli MC, Kaiser R, Wilhelm JL, Kieszak SM (2002). Heat-related mortality during a 1999 heat wave in Chicago. Am J Prev Med.

[b34-ehp-117-1730] O’Neill MS, Zanobetti A, Schwartz J (2003). Modifiers of the temperature and mortality association in seven US cities. Am J Epidemiol.

[b35-ehp-117-1730] O’Neill MS, Zanobetti A, Schwartz J (2005). Disparities by race in heat-related mortality in four US cities: the role of air conditioning prevalence. J Urban Health.

[b36-ehp-117-1730] Schwartz J (2005). Who is sensitive to extremes of temperature? A case-only analysis. Epidemiology.

[b37-ehp-117-1730] Semenza JC, McCullough JE, Flanders D, McGeehin MA, Lumpkin JR (1999). Excess hospital admissions during the July 1995 heat wave in Chicago. Am J Prev Med.

[b38-ehp-117-1730] Semenza JC, Rubin CH, Falter KH, Selanikio JD, Flanders WD, Howe HL (1996). Heat-related deaths during the July 1995 heat wave in Chicago. N Engl J Med.

[b39-ehp-117-1730] Sheridan SC (2007). A survey of public perception and response to heat warnings across four North American cities: an evaluation of municipal effectiveness. Int J Biometeorol.

[b40-ehp-117-1730] Sheridan SD, Dolney TJ (2003). Heat, mortality, and level of urbanization: measuring vulnerability across Ohio, USA. Climate Res.

[b41-ehp-117-1730] Smoyer KE (1998). Putting risk in its place: methodological considerations for investigating extreme event health risk. Soc Sci Med.

[b42-ehp-117-1730] Stafoggia M, Forestiere F, Agostini D, Biggeri A, Bisanti L, Cadum E (2006). Vulnerability to heat-related mortality: a multicity, population-based, case-crossover analysis. Epidemiology.

[b43-ehp-117-1730] Stafoggia M, Forastiere F, Agostini D, Caranci N, de’Donato F, Demaria M (2008). Factors affecting in-hospital heat-related mortality: a multi-city case-crossover analysis. J Epidemiol Community Health.

[b44-ehp-117-1730] Tan J, Zheng Y, Song G, Kalkstein LS, Kalkstein AJ, Tang X (2007). Heat wave impacts on mortality in Shanghai, 1998 and 2003. Int J Biometeorol.

[b45-ehp-117-1730] U.S. Census Bureau (2008). Housing and Household Economic Statistics Division. American Housing Survey (AHS).

[b46-ehp-117-1730] U.S. Department of Health and Human Services (2008). Behaviorla Risk Factor Surveillance System: BRFSS Annual Survey Dat 2002.

[b47-ehp-117-1730] U.S. Department of the Interior (2008). Multi-Resolution Land Characteristics Consortium: National Land Cover Database.

[b48-ehp-117-1730] Vescovi L, Rebetez M, Rong F (2005). Assessing public health risk due to extremely high temperature events: climate and social parameters. Climate Res.

[b49-ehp-117-1730] Whitman S, Good G, Donaghue ER, Benbow N, Shou W, Mou S (1997). Mortality in Chicago attributed to the July 1995 heat wave. Am J Public Health.

